# Molecular Assembly in Block Copolymer-Surfactant Nanoparticle Dispersions: Information on Molecular Exchange and Apparent Solubility from High-Resolution and PFG NMR

**DOI:** 10.3390/polym13193265

**Published:** 2021-09-25

**Authors:** Guilherme A. Ferreira, Watson Loh, Daniel Topgaard, Olle Söderman, Lennart Piculell

**Affiliations:** 1 Institute of Chemistry, University of Campinas (UNICAMP), P.O. Box 6154, Campinas 13083-970, Brazil; wloh@unicamp.br; 2 Division of Physical Chemistry, Lund University, P.O. Box 124, 22100 Lund, Sweden; daniel.topgaard@fkem1.lu.se (D.T.); olle.soderman@fkem1.lu.se (O.S.)

**Keywords:** block copolymer–surfactant complexes, molecular exchange, diffusion NMR

## Abstract

Internally structured block copolymer-surfactant particles are formed when the complex salts of ionic-neutral block copolymers neutralized by surfactant counterions are dispersed in aqueous media. Here, we report the ^1^H NMR signal intensities and self-diffusion coefficients (*D*, from pulsed field gradient nuclear magnetic resonance, PFG NMR) of trimethyl alkylammonium surfactant ions and the poly(acrylamide)-*block*-poly(acrylate) (PAAm-*b*-PA) polyions forming such particles. The results reveal the presence of an “NMR-invisible” (slowly exchanging) fraction of aggregated surfactant ions in the particle core and an “NMR-visible” fraction consisting of surface surfactant ions in rapid exchange with the surfactant ions dissociated into the aqueous domain. They also confirm that the neutral PAAm blocks are exposed to water at the particle surface, while the PA blocks are buried in the particle core. The self-diffusion of the polyions closely agree with the self-diffusion of a hydrophobic probe molecule solubilized in the particles, showing that essentially all copolymer chains are incorporated in the aggregates. Through centrifugation, we prepared macroscopically phase-separated systems with a phase concentrated in particles separated from a clear dilute phase. *D* values for the surfactant and block copolymer indicated that the dilute phase contained small aggregates (ca. 5 nm) of surfactant ions and a few anionic-neutral block copolymer chains. Regardless of the overall concentration of the sample, the fraction of block copolymer found in the dilute phase was nearly constant. This indicates that the dilute fraction represented a tail of small particles created by the dispersion process rather than a true thermodynamic solubility of the complex salts.

## 1. Introduction

Several reports have studied the electrostatic complexation between water-soluble neutral-ionic block copolymers (BCP) and oppositely charged species in solution. The resulting structures are collectively known in the literature as complex coacervate core micelles, C3Ms, featuring a hydrated core and containing the complexed oppositely charged units and a stabilizer shell of neutral water-soluble polymeric blocks [1,2,3,4,5,6].

The most commonly studied C3Ms contain block-copolymers and surfactants. In recent years, stoichiometric (with respect to charge) mixtures of poly(acrylamide)-*block*-poly(acrylate) (PAAm-*b*-PA) and a cationic surfactant have been extensively studied, where the samples were obtained by simply mixing surfactant and BCP stock solutions also containing the “original” simple counterions. The results showed that the resulting aggregates had an average diameter of 50 nm and contained surfactant-rich cores showing no long-range order [7,8,9,10].

Recently, studies of the dispersions of the above-mentioned type of complexes have been extended by employing an alternative methodology based on the complex salt (CS) approach [11]. In the latter approach, stoichiometric block copolymer complex salts (BCPCS) free of simple ions are prepared by simply neutralizing the acrylic acid units of the BCP with the hydroxide salt of the alkyltrimethylammonium cationic surfactant.

By dispersing the freeze-dried BCPCS in water, particles with an average diameter of approximately 300 nm with cores displaying long-range liquid crystalline structures are obtained, as shown in studies by our group in recent years [12,13,14,15]. The colloidal properties and the core structures were studied for particles of varying composition, including additionally added components, such as cosurfactants.

Recently, phase behavior as a function of the water content was investigated in concentrated aqueous BCPCS systems [16]. The latter study showed that the BCPCS did not truly dissolve in water. Mixtures containing more than approximately 50 wt% water phase-separated into a concentrated ordered phase and a dilute phase. Because of the similar densities of the BCPCS and water, a separation into two macroscopic phases was difficult to achieve in normal water, even after extensive centrifugation where a uniformly turbid mixture was obtained. However, when D_2_O was used as a solvent, the centrifugation of water-rich samples resulted in the separation of a dilute bottom phase from a concentrated top phase. DLS results revealed the presence of small (between approximately 10 and 30 nm) particles in the optically clear dilute phases, but no further information on the nature or composition of these particles was obtained.

Regardless of the composition of the BCPCS or the concentration of their dispersions in water, our knowledge on the molecular aggregation of C3Ms is incomplete. Methods such as scattering techniques and electron microscopy are primarily sensitive to the large particles that are formed, but information on the possible presence of small particles and dissociated individual molecules—including the dynamics of molecular exchange processes—is still lacking.

In the present work, we therefore use high-resolution and pulsed field gradient (PFG) ^1^H NMR to study the molecular assembly in BCPCS dispersions. High-resolution ^1^H NMR spectra inform us of the molecular composition of the particles and of the molecular exchange dynamics. PFG NMR methods measure the molecular translational diffusion [17] and have been employed in a variety of surfactants and BCP systems [18,19,20,21,22,23] to estimate the size of their molecular assemblies via the Stokes–Einstein equation. Moreover, information on the possible presence of bound and dissociated surfactant and BCP species can be obtained via their average diffusion coefficients.

## 2. Materials and Methods

Chemicals: The synthesis of the block copolymer PAAm_133_-*b*-PAA_49_ was described earlier [12], while PAAm_422_-*b*-PAA_69_ was a gift from Rhodia (Cranbury, NJ, USA). The subscripts refer to the weight average number of repeating units of each block (Table 1). Both polymers have been used in previous studies by our group, and their characterization is described in references [13,16]. Dodecyl- and hexadecyltrimethylammonium bromide; C_12_TABr and C_16_TABr of 99% purity; D_2_O (99.9% D atoms); and hexamethyldisiloxane, HMDSO, were purchased from Sigma-Aldrich (Saint Louis, MO, USA) and were used as received. Deionized water with a resistivity above 18.2 MΩ·cm^−1^, obtained in a Milli-Q^®^ system, was used in the preparation of the BCPCS.

**Preparation of the BCPCS**: Three BCPCS were prepared following the general procedure described earlier [12,13,14,15,16]. Briefly, the hydroxide form of the surfactants, obtained by an ion-exchange step, was titrated with aqueous solutions of the BCP in the acid form until the equivalence point (pH 8.6–8.9). The resulting mixtures were left overnight at 4 °C, after which their pH values were checked and, when necessary, adjusted to the equivalence point with the corresponding BCP solution. BCPCS in the solid form were isolated from the solution by freeze-drying the mixture and were subsequently kept in a desiccator. The three BCPCS obtained by employing this methodology will be referred to as C_12_S, C_16_S, and C_12_L, respectively, where the terms C_12_ and C_16_ refer to the surfactant alkyl chain length and where S and L refer to the length of the PAAm block in the BCP employed in the synthesis: S for the BCP with a short PAAm block (PAAm_133_-*b*-PA_49_) and L for the BCP with a long PAAm block (PAAm_432_-*b*-PA_70_).

**Preparation of particle dispersions**: Particle dispersions at the desired concentrations were prepared by vortexing the appropriate amounts of solid BCPCS in D_2_O (Vortex-Genie 2 mixer—Scientific Industries, operating at 3200 rpm) for approximately 1 min. The samples thus prepared will be referred to as intact dispersions. All of the intact dispersions for the high-resolution NMR spectra were prepared with a charge concentration of 25 mM of surfactant ion and/or polyion charges. For the self-diffusion measurements, intact dispersions with the final solid concentrations of 0.1, 1.0, or 10.0 wt% were prepared. In addition, centrifuged dispersions were prepared by the centrifugation of 0.1, 1.0, and 10.0 wt% intact dispersions for 24 h at 1000 *g*. The resulting concentrated (upper) and dilute (bottom) phases were collected and studied individually.

**Preparation of particle dispersions with HMDSO**: Particle dispersions labeled with the hydrophobic probe HMDSO [24,25] were obtained by dispersing each of the three investigated BCPCS in D_2_O to achieve 1.0 wt% solid concentration. Pure HMDSO at a probe-to-surfactant molar ratio of 0.01 was added to each dispersion, which was then homogenized with the aid of a vortex mixer. As reference samples, 25 mM micellar solutions of C_12_TABr and C_16_TABr were also prepared and mixed with HMDSO at a probe-to-surfactant molar ratio of 0.01.

**NMR measurements**: High-resolution ^1^H-NMR spectra were obtained using a 400 MHz Bruker Avance spectrometer (Billerica, MA, USA) for the intact dispersions as well as for the solutions of the individual components (surfactants and block copolymers), all of which were at a charge concentration of 25 mM. The signal intensities were quantitatively compared by integrating the corresponding peak areas. The self-diffusion experiments, based on low-resolution ^1^H-NMR spectra, were performed using a Bruker Avance-II 200 (Billerica, MA, USA) operating at a 200 MHz proton resonance frequency equipped with a Bruker diffusion probe with a maximum gradient strength of 9.6 Tm^−1^, unless otherwise stated. The pulsed field gradients (PFGs) were generated in a Bruker DIFF-25 gradient probe driven by a GREAT-40 unit, except where otherwise indicated. These measurements were performed using a maximum gradient strength (*g*) of 4.52 Tm^−1^ with a pulsed gradient duration (*δ*) of 1.0 ms, and the time between the start of two gradient pulses (*∆*) was 20 ms. The stimulated echo (PFG-STE) method was used, with a pulse sequence of 90°-τ_1_–90°-τ_2_–90°-τ_1_–echo [26]. In the PFG-STE experiment, the attenuation of the signal intensity *I* was given by [17]:

(1) $$I=I_{o}exp(-D\gamma^{2}\delta^{2}g^{2}(∆-\frac{1}{3}\delta ))$$

where *I_o_* is the signal intensity in the absence of gradients, *D* is the translational diffusion coefficient, and *γ* is the magnetogyric ratio. All of the measurements were conducted at 25.0 ± 0.5 °C. All of the data processing and the fitting of the diffusion coefficients were achieved using the spectrometer software (Topspin 2.1, Bruker, Billerica, MA, USA). Each diffusion coefficient results from the fit of 12–14 data points (peak areas). The number of significant figures given for the *D* values is based on the reproducibility of the measurements performed in duplicate for selected samples studied at the same conditions.

In principle, any non-overlapping peak from the surfactant, the polyion, or the HMDSO probe can be used to analyze the diffusion of the respective species. Based on a two-state model, assuming that the molecule is either “bound” to the particle or “free” as a non-aggregated unimer in solution, and that the exchange between those two states is fast, the observed diffusion coefficient is given by [17]:

(2) $$D_{obs}=\alpha D_{free}+(1-\alpha )D_{particle}$$

where *D_obs_* is the observed diffusion coefficient of the molecule (surfactant or polyion) in the dispersion, *D_particle_* is the particle diffusion coefficient, *D_free_* is the diffusion coefficient of free surfactant ion or polyion, and *α* is the fraction of free molecules. The unimer diffusion coefficients of the non-aggregated surfactant ions and polyions, corresponding to *D_free_* in Equation (2), were measured in D_2_O and are presented in Table 2.

## 3. Results

### 3.1. High-Resolution ^1^H-NMR Spectra

**Figure 1:**
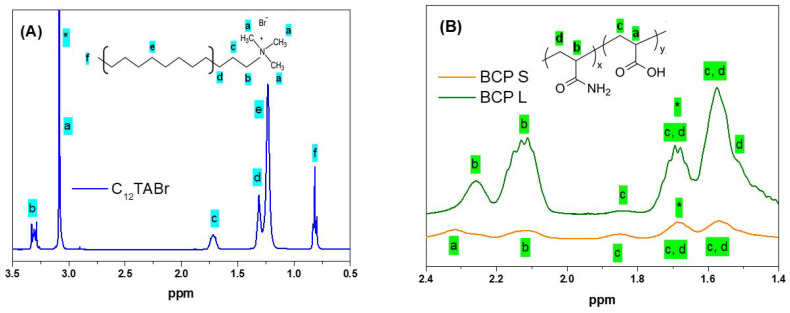
^1^H NMR spectra for (**A**) surfactant and (**B**) block copolymer solutions with labeled peaks according to the chemical structures presented in the inset. The peaks used for diffusion experiments are indicated with asterisks. The terms S and L refer to the length of the PAAm block in the BCP (short and long, respectively). Solutions contain the individual components (surfactants and block copolymers) at a charge concentration of 25 mM, which is above the cmc for the surfactant. Note in (**B**) that since the PA concentration was the nearly the same for the two block copolymer solutions, the intensities of all of the PAAm peaks are much stronger for PAAm_432_-*b*-PAA_70_ (BCP L) than they are for PAAm_133_-*b*-PAA_49_ (BCP S).

High-resolution ^1^H-NMR spectra were obtained for freshly prepared intact C_12_S and C_12_L dispersions as well as for the individual surfactant and block copolymer solutions. All of the samples have the same charge concentration of 25 mM for the surfactant ion and/or polyion charges. Spectra from the solutions of the surfactant and the block copolymers are shown in Figure 1 together with chemical structures and peak assignments. For the surfactant (Figure 1A), the peak in the interval 3.0–3.1 ppm can be attributed to the protons of the trimethyl ((–CH_3_)_3_) group in the surfactant headgroup, whereas the peaks at 3.3, 1.7–1.8, and 1.2–1.3 ppm can be attributed to the methylene (–CH_2_–) groups of the surfactant alkyl chain. The peak at around 0.8 ppm corresponds to the terminal methyl (–CH_3_) group of the surfactant chain.

Peak assignments for the block copolymers are based on published spectra for the homopolymers PAA [27] and PAAm [28]. The two methine (–CH<) groups of the copolymer blocks (Figure 1B) give rise to one partly resolved peak just above 2.3 ppm for PA, which is only visible for PAAm_133_-*b*-PAA_49_, and two peaks at 2.25 and 2.1 ppm for PAAm. The methylene (–CH_2_–) groups of the two blocks give rise to three peaks for each polymer repeat unit, one resolved peak at 1.85 ppm from PA, two overlapping peaks from both repeat units at 1.7 and 1.55 ppm, and a shoulder at 1.45 ppm from PAAm. The relative contributions to the peaks at 1.7 and 1.55 ppm differ between PA and PAAm: the peak at 1.7 is the most intense for PA, whereas the 1.55 ppm peak is the most intense for PAAm.

**Figure 2:**
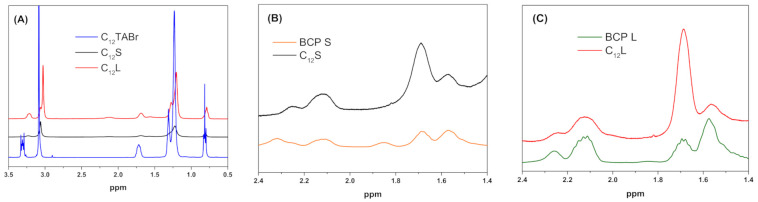
^1^H NMR spectra for intact dispersions compared to spectra for surfactant and block copolymer individual solutions recorded under the same conditions and at the same concentrations as in the dispersions. (**A**) shows comparisons of the surfactant peaks while (**B**,**C**) compare the block copolymer peaks. The term C_12_ denotes the surfactant alkyl chain length whereas S or L refer to the length of the PAAm block in the BCP (short or long, respectively).

The obtained spectra for C_12_S and for C_12_L are displayed at different expansions in the three panels of Figure 2 to highlight the peaks and chemical shifts related to surfactant or the block copolymer, respectively. Each of the panels, A, B and C, also includes a spectrum of the reference surfactant or the block copolymer solution, which were recorded under exactly the same conditions as the BCPCS sample and were plotted at the same magnification along the y axis to allow a direct comparison. Figure 2A shows that the surfactant peaks are broader and are much less intense in the dispersions than in the reference surfactant solution, especially for C_12_S. For the block copolymer, the resolved peaks from PA at 2.3 and 1.85 ppm disappear in the dispersions, while the resolved peaks from PAAm (2.25 and 2.1 ppm) and the overlapping peaks from PA and PAAm are less affected. The disappearance of the PA signals in the C_12_S spectrum was confirmed in a separate experiment. A single experiment (not shown) on C_16_S showed a partial but not total loss of the PA signal intensities compared to the PAAm signal intensities. Figure 2B,C show that there is a contribution from the surfactant to the peak at 1.7 ppm in both dispersions but, nevertheless, the composite signals from the dispersions are much less intense than the signal from the surfactant alone at 1.7 ppm (see Figure 2A).

### 3.2. Compositions of Phase-Separated Samples

**Figure 3:**
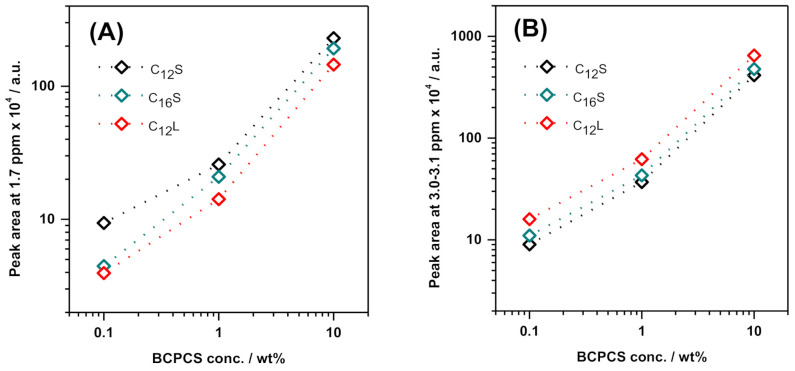
Variation of peak areas at (**A**) 1.7 ppm (composite peak associated with the polyion) and (**B**) 3.0–3.1 ppm (surfactant peak) in the dilute phase obtained in the centrifuged samples as a function of overall initial BCPCS concentration. The lines are guides to the eye. Note the log–log scale. The terms C_12_ and C_16_ denote the surfactant alkyl chain length, and S and L refer to the length of the PAAm block in the BCP (short and long, respectively).

Macroscopically phase-separated dispersions of C_12_S, C_16_S, and C_12_L were prepared by means of the centrifugation of the intact dispersions prepared at 0.1, 1.0, and 10.0 wt%, as described in the Experimental section. In each case, centrifugation resulted in the creation of a clear, dilute bottom phase separated from a concentrated, turbid top phase. The latter phase corresponded to approximately 10% of the sample volume, irrespective of the initial BCPCS concentration. PFG NMR measurements were performed on the separated dilute and concentrated phases as well as on the corresponding intact dispersions. The low-resolution ^1^H NMR spectra obtained in the PFG experiments were also analyzed. The spectra from the dilute phases were quite similar to those from the intact dispersions although, naturally, the intensities in the dilute phases were much weaker. For sensitivity reasons, the integrals from the composite (see above) methylene peak at 1.7 ppm were used as a measure of the BCPCS concentration. Figure 3 shows that for all three of the investigated samples, the BCPCS concentration in the dilute phase was, to a good approximation, proportional to the that of the intact dispersions. For an estimate of the absolute concentrations in the dilute phases, the peak integrals were compared to a calibration curve based on the spectra for the short polymer at different concentrations. From this comparison, we conclude that in all of the dilute phases, the concentration of BCP was in the range 5–10% of the concentration in the intact dispersion. Note that, due to intensity losses (see Section 3.1 above), the spectra from the intact dispersions were not suitable as a reference to measure BCPCS concentration. However, since the dilute phases contained only small particles (see below), it was assumed that their spectra did not suffer any intensity loss.

### 3.3. Self-Diffusion Measurements

**Figure 4:**
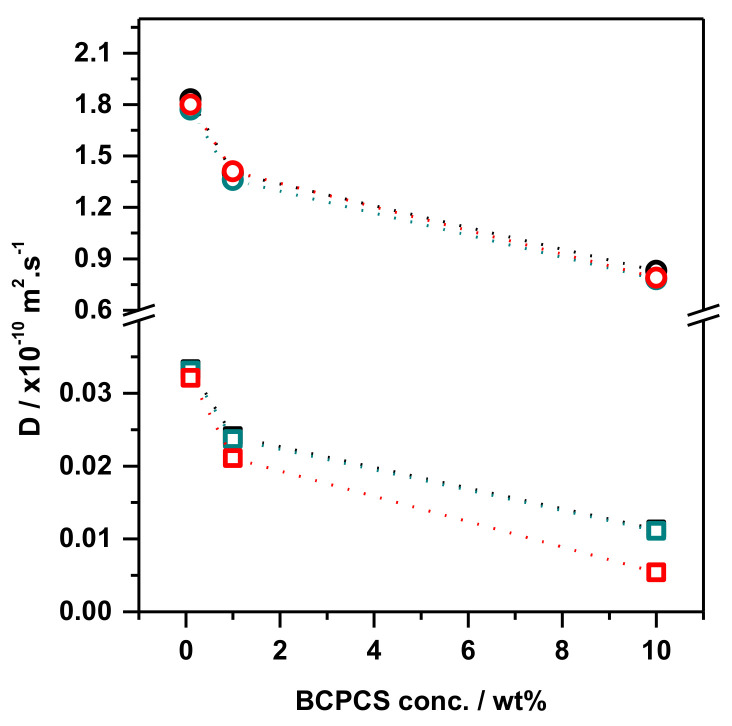
Self-diffusion coefficient values (*D*) as a function of BCPCS concentration in intact samples. Symbols denote the C_12_TA^+^ ion (**○**) and polyion (**□**) in C_12_S; the C_16_TA^+^ ion (**○**) and polyion (**□**) in C_16_S; and the C_12_TA^+^ ion (**○**) and polyion (**□**) in C_12_L. The lines are guides for the eye. The terms C_12_ and C_16_ refer to the surfactant alkyl chain length, whereas S and L refer to the length of the PAAm block in the BCP (short and long, respectively).

Self-diffusion measurements were performed on both the intact and phase-separated (centrifuged) samples. The surfactant ion self-diffusion was analyzed from the methyl proton signal of the trimethylammonium headgroup in the ^1^H NMR spectra (peak at 3.1 ppm—Figure 1A). The BCP diffusion coefficient was obtained from the methylene group signal at 1.7 ppm, which, again, was for sensitivity reasons (Figure 1B). Figure 4 shows the obtained self-diffusion coefficients for the ionic species in all of the investigated intact samples.

In a control experiment, freshly prepared intact samples at 1 wt% solid concentration were labeled with the hydrophobic probe HMDSO at a probe-to-surfactant molar ratio of 0.01 in order to reach approximately one probe molecule per micelle, a protocol used in previous studies of similarly labelled micellar surfactant solutions [23,24]. A comparison of the low-resolution ^1^H NMR spectra obtained for the HMDSO-labeled dispersions and the corresponding surfactant solutions (Figure S1) confirms that the surfactant signal intensities are significantly reduced in the dispersions. Table 3 shows the obtained self-diffusion coefficient values for the surfactant ions, polyions, and hydrophobic probe for the three investigated particle dispersions. For each sample, the HMDSO and polyion diffusion coefficients differ by less than 10%.

**Figure 5:**
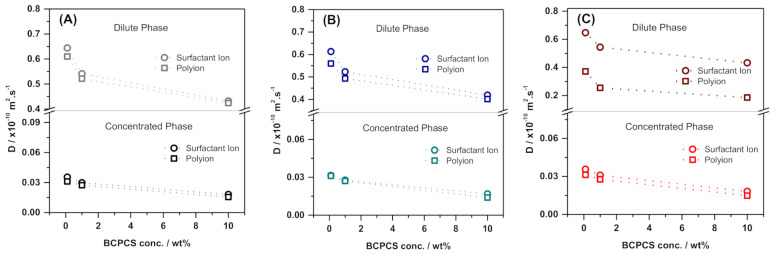
Self-diffusion coefficients (D) for surfactant ions and polyions in the concentrated and dilute phases obtained for centrifuged samples at different (overall) initial concentrations for (**A**) C_12_S, (**B**) C_16_S, and (**C**) C_12_L. The lines are guides for the eye. The terms C_12_ and C_16_ refer to the surfactant alkyl chain length, whereas S and L refer to the length of the PAAm block in the BCP (short and long, respectively).

The self-diffusion coefficients of the surfactant ions and polyions measured in the concentrated and dilute phases of the centrifuged samples obtained from BCPCS dispersions at different overall concentrations are displayed in Figure 5.

## 4. Discussion

### 4.1. High-Resolution ^1^H NMR Spectra

**Figure 6:**
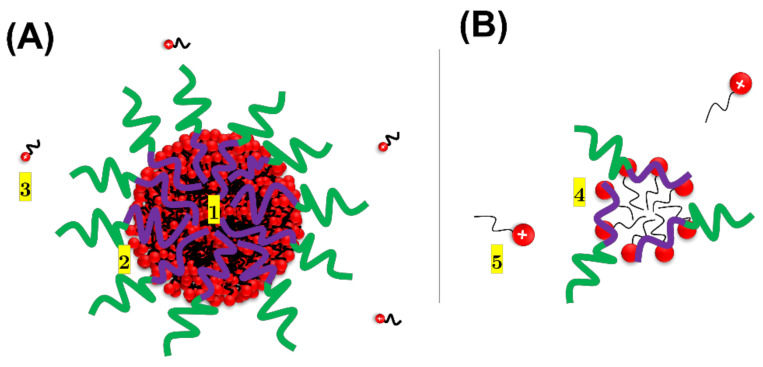
Schematic illustration of distinguishable populations of surfactant ions in BCPCS particle dispersions: (**A**) large particles (predominating in intact samples and concentrated phases) with a dense liquid crystalline core formed by surfactant micelles surrounded by the polyion chains; (**B**) small aggregates (predominating in dilute phases) formed by aggregated surfactant ions neutralized by a few polyion chains. In (**A**), the sites labeled as 1, 2, and 3 correspond to the core, surface, and free (dissociated) surfactant ions, respectively. The exchange between the core and surface surfactant ions is slow, whereas the exchange between the surface and free surfactant ions is fast. In (**B**), the sites labeled as 4 and 5 correspond to the surface and free surfactant ions, which undergo a fast exchange. In both cases, the aggregate shape is arbitrarily depicted as spherical. Green, purple, red, and black segments denote the PAAm block, the PA block, the surfactant headgroup, and the surfactant alkyl chain, respectively.

High-resolution ^1^H NMR spectra (Figure 2) obtained for intact BCPCS samples at 1.0 wt% display characteristic signals belonging to both surfactant and block copolymers. A significant loss of signal intensity is consistently seen for all of the surfactant peaks compared to the pure surfactant solution (Figure 2A), which indicates that only a portion of the surfactant content is seen in the spectra and contributes to the measured self-diffusion coefficients. The spectra obtained for the micellar solutions and the particle dispersions labeled with HMDSO also confirm a substantial loss of signal intensity for the surfactant peaks relative to HMDSO (Figure S1). This indicates that the surfactant molecules in the particles fall into two categories, as illustrated in Figure 6.

The “NMR visible” surfactant ions consist of “free” surfactant unimers, which are dissociated into the water domain, and “surface” surfactant ions, which are situated sufficiently close to the particle–water interface in order to undergo rapid exchange with the free surfactant ions. The timescale for rapid exchange is milliseconds, which was determined by the PFG experiment. The “NMR invisible” surfactant ions, by contrast, are surfactant molecules in the interior of the particles, which exchange too slowly with the surface and free fractions to contribute to a visible high-resolution signal. Instead, the invisible fraction gives rise to a separate signal, which is too broad to be detected in the high-resolution NMR experiment. The cores of the large BCPCS particles are known to contain surfactant ion aggregates arranged in ordered liquid crystalline structures, resulting in slow surfactant diffusion coefficients and very broad spectra [19]. The ^1^H NMR linewidth is determined by molecular dynamics that average the dipolar spin–spin coupling to zero. As explained in more detail in reference [19], the reorientation of the surfactant aggregates in the concentrated liquid crystalline structure of the particle core is severely hindered, and the relatively rapid diffusion of the surfactant ions over the surfaces of the surfactant aggregates does not suffice to average the ^1^H-^1^H dipolar coupling to zero since the aggregates are anisometric. The diffusional exchange of surfactant ions between the aggregates of the uncorrelated orientations is also slow.

Regarding the block copolymer signals (Figure 2B,C), the well-resolved signals from PAAm are only moderately affected by incorporation in the particles, which is consistent with the a priori notion that the PAAm blocks are all situated at the surface of the particle in a hydrated shell, where they experience considerable motional freedom. By contrast, the high-resolution PA signals from the particles have disappeared entirely for C_12_S (Figure 2B,C) and partially for C_16_S. This suggests that the PA chains are largely buried in the particle core, where they reorient much more slowly and anisotropically, giving rise to very broad signals, similar to the NMR-invisible surfactant ions in the core.

### 4.2. Self-Diffusion Measurements

The self-diffusion coefficients for polyions in intact samples (Figure 4) and in the concentrated phase of the centrifuged samples (Figure 5A–C) were similar, indicating that they predominantly contained particles of similar sizes. The close similarity (Table 3) between the polyion diffusion and the diffusion of HMDSO, which was quantitatively incorporated in the surfactant micelles inside the BCPCS particles, confirms that the polyion diffusion essentially measures the particle diffusion.

A striking feature seen in Figure 4 is the very rapid surfactant diffusion in intact dispersions. For the 1 wt% dispersions of the three BCPCS that were investigated, the measured surfactant diffusion was approximately one third of the unimer diffusion measured in aqueous surfactant solutions below the cmc (Table 2). At this stage, we are unable to quantitatively explain such a rapid surfactant diffusion, which was reproducibly observed by measurements of several preparations, which also used different spectrometers. To qualitatively understand this result, we recall from the previous Section 4.1 that only a fraction of the surfactant ions is NMR visible, and it is only for this pool of surfactant ions that the fast exchange condition, resulting in Equation (2) above, applies. The rapidly exchanging surfactant ions must reside on the particle surfaces, but we have no independent information on the surface-to-volume ratio in the polydisperse particle dispersions. Conductivity measurements (see Supporting Information for details) show, however, that the concentration of free surfactant ions is very low (approximately 1 mM for 1 wt% C_12_S or C_16_S) in the intact particle dispersions (Figure S2).

A further complicating factor in the study of the intact particle dispersions is our finding that a slow creaming process occurs for BCPCS dispersions in D_2_O. The density difference between the particles and D_2_O, which allows particles to be separated by centrifugation, also leads to a continuous net transport of preferentially larger particles towards the top of an intact sample under gravity. We could observe the creaming process by monitoring the NMR signal intensities and the self-diffusion coefficients for the samples left unstirred in the spectrometer over 50 h, as reported in detail in the Supplementary Materials (see Figures S3 and S4 and the accompanying text). The signal intensity decreased with time, indicating a decreasing concentration of BCPCS particles in the lower part of the NMR tube, which was probed in the NMR experiment. Simultaneously, the recorded average particle diffusion increased with time, indicating that the remaining particles had a smaller average size compared to the full distribution. Significant creaming already occurred at the timescale of hours. However, a gentle mixing of an aged sample after 3 days restored the properties of a fresh dispersion (Table S2 and Figure S5), confirming that creaming, rather than changes in the particle size distribution as such, was indeed the mechanism responsible for the time-dependent phenomena.

One may ask to what extent the particular NMR features found here for surfactant ions in intact dispersions, namely the reduced proton signal intensity and the apparent rapid diffusion, are dependent on the complex salt approach used to produce the dispersions. To check this, we made additional measurements on dispersions of C_12_TABr with the sodium salts of the two BCP prepared by the conventional mixing protocol in stoichiometric proportions. The conventionally prepared dispersions indeed displayed the same features as the intact dispersions: a much-decreased surfactant signal intensity (Figure S6) coupled with a very rapid surfactant ion diffusion coefficient (Table 4). Table 4 also shows that the polyion diffusion, which corresponds to the particle diffusion, was significantly more rapid for the conventionally prepared particles, corresponding to a smaller hydrodynamic radius of approximately 50 nm. The difference in size between the differently prepared particles was confirmed by DLS measurements (Table 4) and was in line with previous data from scattering and microscopy techniques for similarly prepared particle dispersions [7,8,9,10].

We will now turn to the centrifuged dispersions of all three BCPCS. In the separated concentrated phases of all three BCPCS, the observed self-diffusion coefficients for the polyion and surfactant ion were quite similar, with a surfactant ion diffusion that was only slightly more rapid than the polyion ion diffusion. This result supports the notion that surfactant ions are essentially quantitatively incorporated in large BCPCS particles. Our main objective when studying centrifuged samples was, however, to obtain more detailed information on the small particles remaining in the dilute phases of these samples.

Clearly, for a truly binary mixture of water with a BCPCS containing strictly monodisperse polyions and surfactant counterions, the concentration of BCPCS detected in the dilute phase would be independent of the overall concentration of the system at thermodynamic equilibrium since it would then simply correspond to the solubility of the BCPCS in water. One conceivable origin of a constant soluble polymer fraction in the dilute phase could thus be the existence of a molecularly distinct fraction of polymer that is unable to form complexes with the surfactant due to a low or vanishing content of charged units. However, this explanation is ruled out in our case by the finding from NMR that the dilute phase also contains surfactant ions and that the diffusion coefficients of these surfactant ions show that they are largely associated with the polyions. The surfactant ion diffusion coefficients are much lower than the respective unimer diffusion coefficients (Table 2), and their concentration dependence closely follows that of the polyions (Figure 4).

The polyion diffusion in the dilute phases, in turn, varies, as expected from the molecular masses of the respective polyions; that is, it increases in the sequence *D*(C_12_L) < *D*(C_16_S) ≈ *D*(C_12_S). For all of the particles the polyion diffusion coefficients show a rather strong concentration dependence, and for the lowest initial concentration, they, in fact, approach the diffusion coefficients of the respective single polyions (compare data in Figure 4 and Table 2). Collectively, the data thus indicate that the dilute systems contain small BCPCS complexes, each containing aggregated surfactant ions and at most a few polyions, the latter being of a size that is comparable to the average size of the BCP used in the complex formation (Figure 6).

In conclusion, the properties of the polyions in the dilute phase complexes do not seem to differ significantly from the average properties of the respective polyion samples. We must therefore consider a different explanation for the existence of a constant fraction of apparently soluble BCPCS particles. We suggest that the small aggregates in the dilute phase simply represent a fraction of very small aggregates from a wide distribution of aggregate sizes. We must then assume that a reproducible size distribution is created during the dispersion of the BCPCS in water, that the distribution is only weakly dependent on the initial concentration of BCPCS, and that the created particles are stable in time. The last conclusion is supported by the fact that the original NMR intensities and average diffusion coefficients were obtained in samples subjected to three days of creaming after a gentle mixing of the samples. (Figure S5 and Table S1).

**Figure 7:**
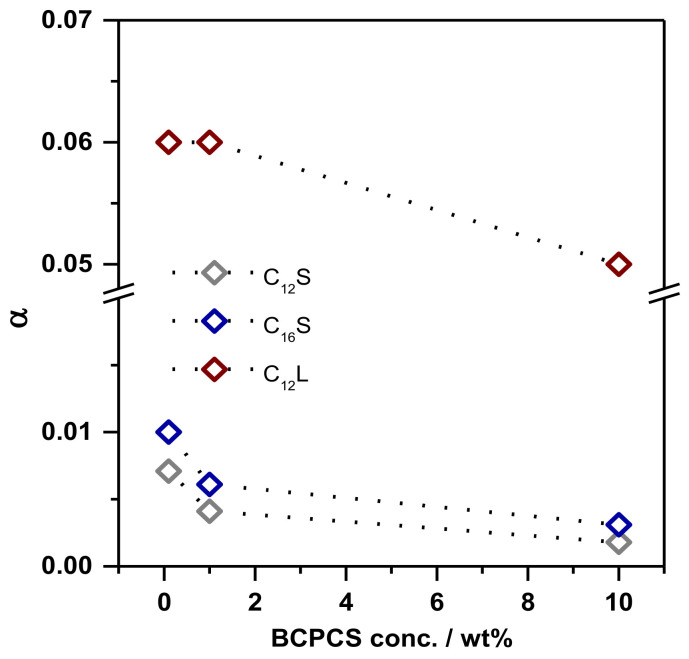
Fraction of dissociated surfactant ions (*α*) as a function of BCPCS concentration in the dilute phases obtained by centrifuging BCPCS samples at different overall (initial) concentrations. The lines are guides for the eye. The terms C_12_ and C_16_ refer to the surfactant alkyl chain length, and S and L refer to the length of the PAAm block in the BCP (short and long, respectively).

In any case, the complexes of the dilute phases are sufficiently small enough to justify the assumption of rapid exchange and narrow NMR linewidths, so the use of Equation (2) should be legitimate for these systems. Hence, the equation has been used to calculate the dissociated fraction of the surfactant ions (α), and the results are shown in Figure 7.

In the calculations, we assumed that *D_particle_* equals the measured diffusion coefficient of the polyion in each sample. For C_12_S and C_16_S, the fractions of the free surfactant ions are very small, confirming that the latter are in fact self-assembled into micelles complexed to the polyions, as sketched in Figure 6B, which is in agreement with experimental evidence on the nature of polyion–surfactant ion complexation in dilute solution [5,6]. Interestingly, *α* is significantly larger for C12L, indicating that the presence of a large neutral block on the polyion may partially hinder the micellization of the surfactant at the polyion.

## 5. Conclusions

The dispersion of BCPCS in water by vortexing generates a rather wide but stable and reproducible size distribution of BCPCS particles. When D_2_O rather than H_2_O is used as the solvent, the particles slowly separate according to size by creaming.

In particular, the distribution includes a fraction of small aggregates that do not separate out when the dispersion is subjected to centrifugation but remain in solution as one or a few (depending on concentration) block copolymer chains attached to one or a few surfactant ion micelles. The concentration of such small aggregates in the dilute phase increases in proportion to the overall content of BCPCS, but their molecular components, the BCP in particular, do not differ measurably in composition from the bulk average. No evidence is thus found for a true thermodynamic solubility of BCPCS; the apparent solubility in the dilute phase results from the formation of small kinetically stable particles.

The much larger BCPCS particles that dominate the full distribution contain a surface pool of surfactant ions in rapid exchange with a small fraction of surfactant ions that have dissociated from the particles, separated from a slowly exchanging pool of surfactant ions inside the particle cores. This picture holds also for particles that have been conventionally prepared by mixing surfactant solutions with block polyion, including their respective simple counterions.

The neutral PAAm blocks are highly mobile, indicating that they extend out into the aqueous phase. By contrast, the polyacrylate blocks are largely buried in the particle cores. The fraction of block polyions dissociated from the particles is essentially zero.

## Figures and Tables

**Table 1:** Weight average molar mass of the blocks and dispersity (PDI) of the PAAm-*b*-PAA block copolymers (in the acid forms) employed in this work.

Block Copolymer	PAAm/g·mol^−1^	PAA/g·mol^−1^	PDI ^a^
PAAm_133_-*b*-PAA_49_	9415	3500	2.1 ^b^
PAAm_432_-*b*-PAA_70_	30,680	5000	1.6 ^c^

^a^ PDI = M_w_/M_n_, as determined by gel permeation chromatography. ^b^ According to ref. [16]. ^c^ According to ref. [10].

**Table 2:** Unimer self-diffusion coefficients of surfactant ions and polyions obtained using the indicated peaks in ^1^H NMR spectra.

Species	*D*/× 10^−10^ m^2^.s^−1^	Peak Position/ppm
C_12_TA^+^^a^	4.86	3.1
C_16_TA^+^^b^	4.14	3.1
PAAm_133_-*b*-PAA_49_ ^c^	0.66	1.7
PAAm_432_-*b*-PAA_70_ ^d^	0.31	1.7

^a^ In 10 mM C_12_TABr solution (cmc = 12 mM). ^b^ In 0.7 mM C_16_TABr solution (cmc = 1 mM). ^c^ In 10 mM, based on AA monomer, PAAm_133_-*b*-PAA_49_ solution. ^d^ In 10 mM, based on AA monomer, PAAm_432_-*b*-PAA_70_ solution.

**Table 3:** Self-diffusion coefficients (D) for surfactant ions, polyions, and HMDSO in intact samples at 1 wt% BCPCS.

BCPCS	Species	*D*/m^2^.s^−1^
C_12_S	C_12_TA^+^ PAAm_133_-*b*-PA_49_ HMDSO	1.43 × 10^−10^ 2.33 × 10^−12^ 2.12 × 10^−12^
C_12_L	C_12_TA^+^ PAAm_432_-*b*-PA_70_ HMDSO	1.38 × 10^−10^ 2.10 × 10^−12^ 1.90 × 10^−12^
C_16_S	C_16_TA^+^ PAAm_133_-*b*-PA_49_ HMDSO	1.32 × 10^−10^ 2.35 × 10^−12^ 2.18 × 10^−12^

**Table 4:** Self-diffusion coefficients for surfactant ions (D_surf_) and polyions (D_pol_) in dispersions at 25 mM of charge concentration: comparing dispersions prepared by the complex salt approach to those prepared by a conventional protocol. The table also includes hydrodynamic radii (R_H_) obtained from dynamic light scattering (DLS) and from the polyion self-diffusion coefficients using the Stokes–Einstein relation. Self-diffusion measurements performed in a 400 MHz Bruker Avance spectrometer.

Sample	*D_surf_* × 10^−10^/m^2^·s^−1^	*D_pol_* × 10^−12^/m^2^·s^−1^	*R_H_* (DLS)/nm ^c^	*R_H_* (*D_pol_*)/nm ^d^
C_12_S ^a^	1.60	3.00	120	131
C_12_L ^a^	1.60	2.40	130	164
C_12_S ^b^	1.40	7.00	50	56
C_12_L ^b^	1.70	6.30	55	63

^a^ Samples prepared by the complex salt approach, as described in Experimental section. ^b^ Samples of C_12_TABr with the sodium salts of the two BCP prepared by conventional mixing in stoichiometric proportions. ^c^ Determined by dynamic light scattering measurements performed as in ref. [13]. ^d^ Calculated from the polyion self-diffusion coefficient using the Stokes–Einstein equation (see refs. [22,23]).

## Data Availability

Not applicable.

## Supplementary Materials

The following are available online at https://www.mdpi.com/article/10.3390/polym13193265/s1, Figure S1: Low-resolution ^1^H NMR spectra for samples labeled with the hydrophobic probe HMDSO. (A) micellar solutions of C_12_TABr and C_16_TABr at 25 mM. (B) BCPCS *intact* dispersions at 1.0 wt%. In all cases, the surfactant-to-HMDS molar ratio is 0.01. For both cases, * denotes the peak used to measure the self-diffusion of HMDSO, Figure S2: (A). Conductivity (κ) of C_12_TABr solutions as a function of C_12_TA^+^ surfactant ion concentration (open symbols). The horizontal lines denote the conductivity of the intact samples of C_12_S (red) and C_12_L (blue) at 1 wt%. (B). Conductivity (κ) of the C_16_TABr solutions as a function of the C_16_TA^+^ surfactant ion concentration (open symbols). The horizontal green line denotes the conductivity of the intact C_16_S sample at 1 wt%, Figure S3: Self-diffusion coefficients (D) for surfactant ion (circles) and polyion (squares) in an intact 1.0 wt. % C_12_S as a function of time for a sample prepared in D_2_O. Red points refer to a sample in which the solvent is a 30:70 H_2_O:D_2_O mixture, Figure S4: Low-resolution ^1^H NMR spectra for an intact 1.0 wt. % C_12_S sample as a function of time of sample preparation in (A) D_2_O; (B) 30:70 H_2_O:D_2_O mixture, Figure S5: Low-resolution ^1^H NMR spectra for a 1.0 wt. % intact C_12_S dispersion 3 days after sample preparation followed by redispersion in (A) D_2_O; (B) 30:70 H_2_O:D_2_O mixture. For comparison purposes, spectra obtained at 0 h and 50 h are presented again for both samples, Figure S6: High-resolution ^1^H NMR spectra obtained for C_12_S and C_12_L nanoparticle dispersions prepared by the conventional protocol of mixing of the aqueous solutions of surfactant salt and sodium salt of BCP at a charge ratio of 1. The molar charge concentration for each species is equal to 25 mM, Table S1: Self-diffusion coefficients obtained for surfactant ion (*D_surf_*) and polyion (*D_pol_*) in a 1 wt% intact C_12_S sample prepared in D_2_O and in a mixture of H_2_O:D_2_O at different time intervals (t).
