# Supplementary material for: Molecular Assembly in Block Copolymer-Surfactant Nanoparticle Dispersions: Information on Molecular Exchange and Apparent Solubility from High-Resolution and PFG NMR
Source: Polymers (Basel). 2021 Sep 25;13(19):3265. doi: 10.3390/polym13193265 (PMC8512401; doi:10.3390/polym13193265)
Supplement: Supplementary file 1 [file polymers-13-03265-s001.zip › polymers-1378708-supplementary.pdf]

Supporting Information

Molecular assembly in block copolymer-surfactant nanoparticle dispersions: Information on molecular exchange and apparent solubility from high resolution and PFG NMR.

Guilherme A. Ferreira[1*#], Watson Loh[1], Daniel Topgaard[2], Olle Söderman[2], Lennart Piculell[2*]

[1]*Institute of Chemistry, University of Campinas (UNICAMP), P.O. Box 6154, 13083-970, Campinas – SP, Brazil.*
[2]*Division of Physical Chemistry, Lund University, P.O. Box 124, S-221 00, Lund, Sweden*

* *ferreira.guilherme@ufba.br*
* *lennart.piculell@fkem1.lu.se*

[#]*Current Address: Department of Physical Chemistry, Institute of Chemistry, Federal University of Bahia, Salvador - BA, Brazil.*

Particles labeled with HMDSO

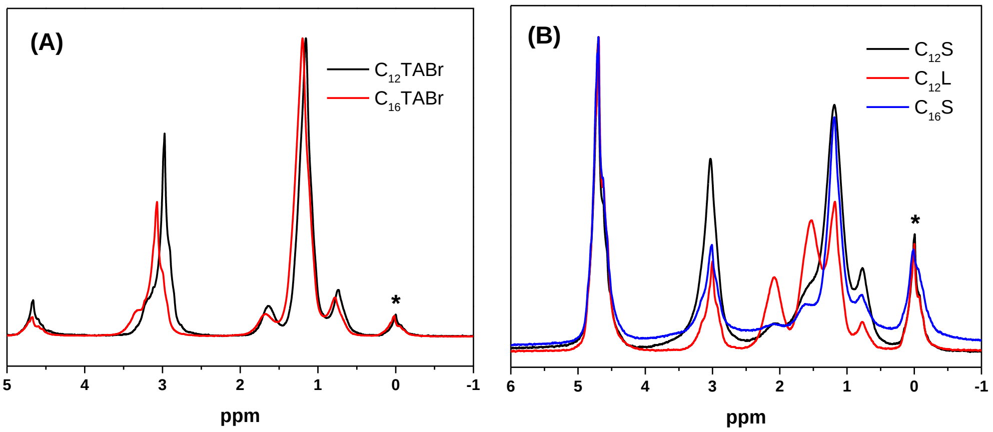

Figure S1. Low resolution ^{1}H NMR spectra for samples labeled with the hydrophobic probe HMDSO. (A) micellar solutions of C_{12}TABr and C_{16}TABr at 25 mM. (B) BCPCS *intact* dispersions at 1.0 wt%. In all cases, the surfactant-to-HMDS molar ratio is 0.01. For both cases, * denotes the peak used to measure the self-diffusion of HMDSO.

Conductivity

Aqueous solutions of C_{12}TABr and C_{16}TABr surfactant, as a function of surfactant ion concentration were prepared, and their conductivity was measured in a digital conductivity meter. The results are displayed in Figure S2. The conductivity of the C_{12}S, C_{16}S and C_{12}L *intact* samples at 1 wt% is also displayed in the same figure (horizontal lines). If one assumes $\alpha = 0.3$ (see text in the main article), the concentration of dissociated surfactant ions in the solution would be around 6 mM, 5.4 mM and 4 mM for C_{12}S, C_{16}S and C_{12}L, respectively (based on the molecular weight of a BCPCS unimer - diblock copolymer chain with its associated surfactant counterions). Clearly, the results in Figure S2 show that the measured conductivities of *intact* samples at 1 wt% corresponds to much lower concentrations of free surfactant ions.

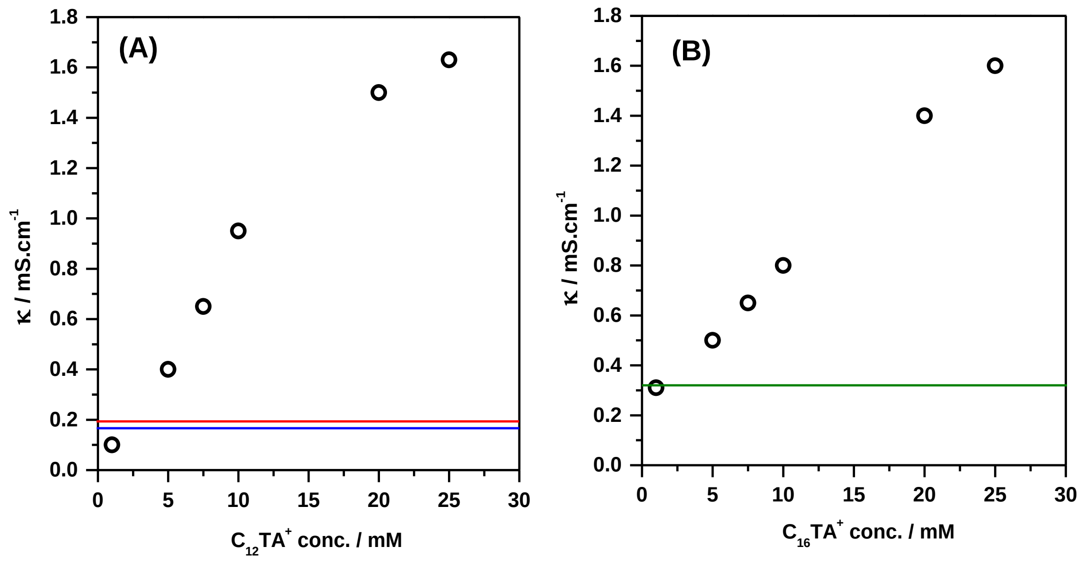

Figure S2. **(A).** Conductivity (κ) of $C_{12}TABr$ solutions as a function of $C_{12}TA^+$ surfactant ion concentration (open symbols). The horizontal lines denote the conductivity of $C_{12}S$ (red) and $C_{12}L$ (blue) *intact* samples at 1 wt%. (B). Conductivity (κ) of $C_{16}TABr$ solutions as a function of $C_{16}TA^+$ surfactant ion concentration (open symbols). The horizontal green line denotes the conductivity of $C_{16}S$ *intact* sample at 1 wt%.

Self-diffusion measurements as a function of time of sample preparation

A 1 wt% $C_{12}S$ *intact* sample was prepared in D_2O and in a mixture of (30:70) H_2O:D_2O. Soon after preparation, the samples were analyzed by PFG NMR at time 0 (few minutes after sample preparation) and at different time intervals. The obtained D values for both surfactant ion and polyion are presented in Figure S3. Clearly, the obtained values increase with time. The low-resolution spectra used for self-diffusion measurements in D_2O were also registered as a function of time and are presented in Figure S4. After 3 days, the samples were gently mixed and measured again. The results (Table S1 and Figure S5) shows that the obtained self-diffusion coefficients and line shapes are remarkably similar to the ones obtained at time 0.

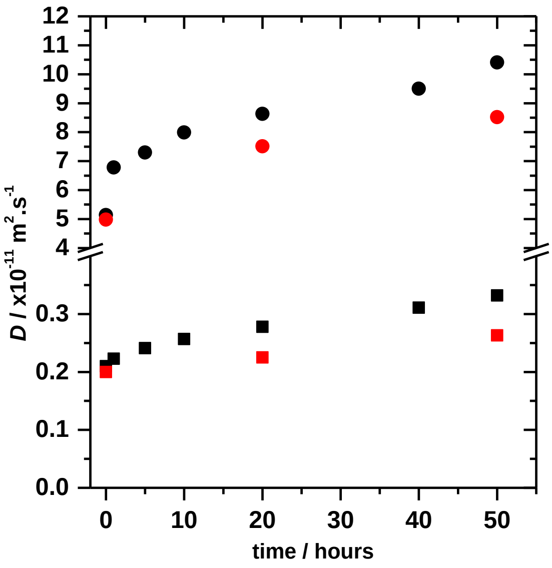

Figure S3. Self-diffusion coefficients (D) for surfactant ion (circles) and polyion (squares) in an *intact* 1.0 wt. % $C_{12}S$ as a function of time for a sample prepared in D_2O. Red points refer to a sample in which the solvent is a 30:70 H_2O:D_2O mixture.

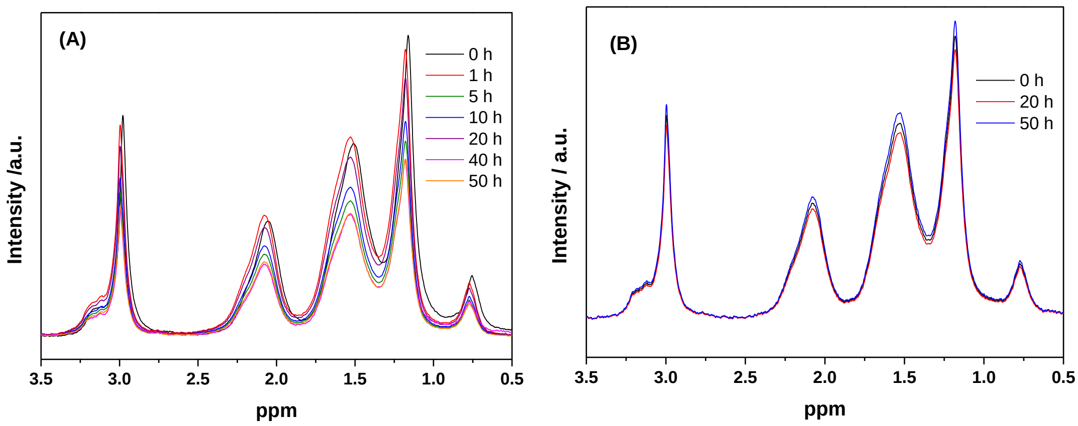

Figure S4. Low resolution ^{1}H NMR spectra for an *intact* 1.0 wt. % $C_{12}S$ sample as a function of time of sample preparation in (A) D_2O; (B) 30:70 H_2O:D_2O mixture.

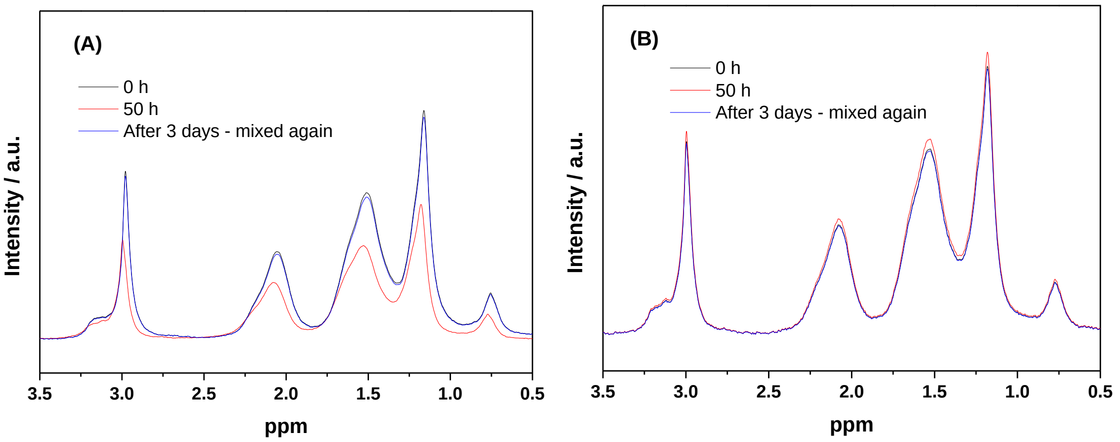

Figure S5. Low resolution ^{1}H NMR spectra for a 1.0 wt. % $C_{12}S$ *intact* dispersion after 3 days after sample preparation, followed by redispersion, in (A) D_2O; (B) 30:70 H_2O:D_2O mixture. For comparison purposes, spectra obtained at 0 h and 50 h are presented again for both samples.

Table S1. Self-diffusion coefficients obtained for surfactant ion (D_{surf}), polyion (D_{pol}) in a 1 wt% $C_{12}S$ *intact* sample prepared in D_2O and in a mixture of H_2O:D_2O at different time intervals (t).

**t / h**	**D_{surf} / $m^2.s^{-1}$**		**D_{pol} / $m^2.s^{-1}$**	
	D_2O	H_2O:D_2O	D_2O	H_2O:D_2O
**0**	0.51×10^{-10}	0.50×10^{-10}	2.10×10^{-12}	2.00×10^{-12}
**50**	1.04×10^{-10}	0.85×10^{-10}	3.30×10^{-12}	2.60×10^{-12}
**3 days – mixed again**	0.52×10^{-10}	0.50×10^{-10}	2.30×10^{-12}	2.20×10^{-12}

$C_{12}S$ and $C_{12}L$ nanoparticle dispersions prepared by the conventional protocol of mixing

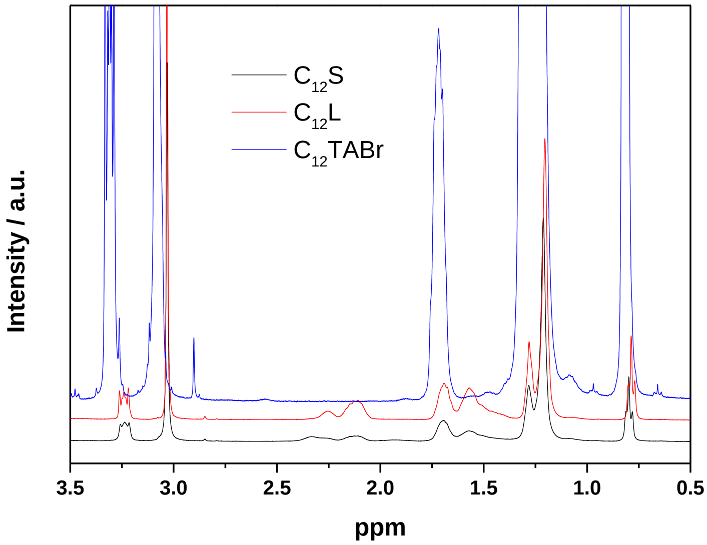

Figure S6. High resolution ^{1}H NMR spectra obtained for $C_{12}S$ and $C_{12}L$ nanoparticle dispersions prepared by the conventional protocol of mixing of aqueous solutions of surfactant salt and sodium salt of BCP at a charge ratio of 1. Spectrum for $C_{12}TABr$ solution is also shown. Molar charge concentration for each specie equal to 25 mM.
